# When to start vasopressin in septic shock: the strategy we propose

**DOI:** 10.1186/s13054-022-04001-4

**Published:** 2022-05-06

**Authors:** Philippe Guerci, Thibaut Belveyre, Nicolas Mongardon, Emmanuel Novy

**Affiliations:** 1 Department of Anesthesiology and Critical Care Medicine, Institut Lorrain du Coeur Et Des Vaisseaux, University Hospital of Nancy, Rue du Morvan, 54511 Vandoeuvre-les Nancy, France; 2 INSERM U1116, DCAC, University of Lorraine, Nancy, France; 3 Service d’Anesthésie-Réanimation Chirurgicale, DMU CARE, DHU A-TVB, Assistance Publique-Hôpitaux de Paris (AP-HP), Hôpitaux Universitaires Henri Mondor, 94010 Créteil, France; 4 Faculté de Santé, Université Paris Est Créteil, 94010 Créteil, France; 5 U955-IMRB, Equipe 03 “Pharmacologie Et Technologies Pour Les Maladies Cardiovasculaires (PROTECT)”, Inserm, Univ Paris Est Créteil (UPEC), Ecole Nationale Vétérinaire d’Alfort (EnVA), 94700 Maisons-Alfort, France; 6 SIMPA, UR 7300, University of Lorraine, 54000 Nancy, France

## Comment

The indications for arginine vasopressin (AVP) are still debated. Wieruszewski and Khanna recently suggested to evolve from the classical stepwise approach towards an early multimodal vasopressor therapy strategy [1]. As mentioned by the authors, the adequate timing of initiation of a second vasopressor remains a challenge. The Surviving Sepsis Campaign (SSC) 2021 proposes to start AVP in septic shock when the dose of norepinephrine (NE) base is in the range of 0.25–0.5 µg/kg/min [2]. Basing the decision on a threshold dose in vasodilatory shock is an easy bedside rule but has some flaws. First, Leone et al*.* raised a warning about NE chemical formulation as referred to NE bitartrate/tartrate versus base [3]. Indeed, a consensual NE formulation should be considered when administration is based on a dose threshold, because NE doses as NE tartrate are twice as high as those expressed as NE base, resulting in a 1–4 ratio threshold according to the SCC recommendations. Second, as noticed by Wieruszewski and Khanna, the pharmacologic response to NE should be characterized individually [1]. NE pharmacokinetics are best described using a one-compartment linear model and follows a log dose–response curve. This questions the interest of a weight-based threshold. Interestingly, several recent publications from experts report NE dose in µg/min, independently of weight [4]. The weight-based strategy could lead to a delay of AVP initiation in some patients, particularly in the growing proportion of obese critically ill patients. High doses of NE at AVP initiation may be associated with an increased risk of mortality [4]. Also, AVP doses are not adjusted for weight but rather used at a fixed dosage (up to 0.04 units/min).

**Fig. 1:**
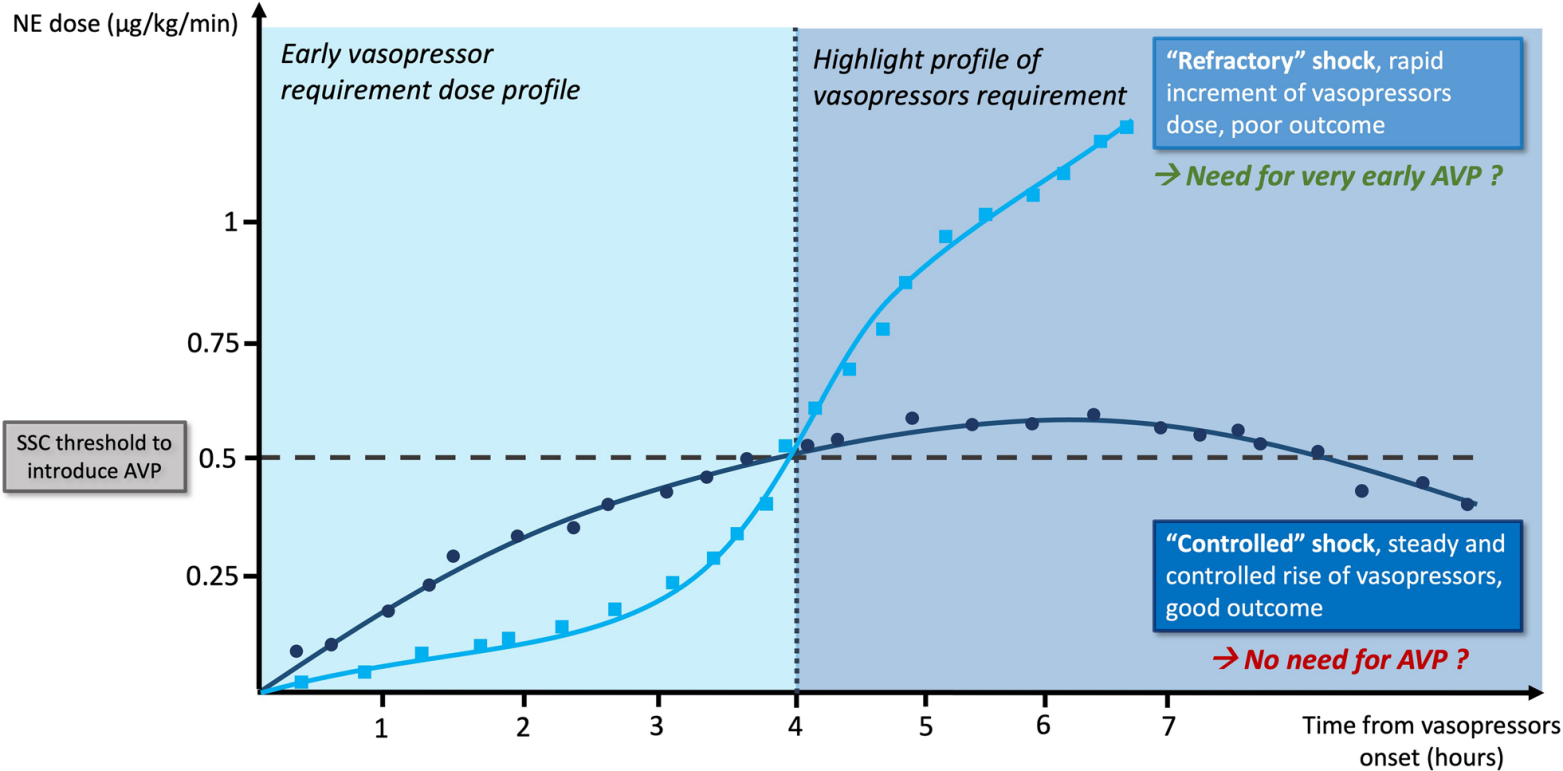
Norepinephrine requirement dose profiles over time. Both curves were obtained after modelling of NE evolution over time of a “controlled” shock (dark blue line with dots) and a “refractory” shock (clear blue line with squares). Both profiles overshot the threshold of 0.5 µg/kg/min of NE dose, but only the “refractory” shock profile would benefit the most of a very early AVP infusion (NE < 0.25 µg/kg/min). AVP: Arginine vasopressin; NE: norepinephrine; and SSC: Surviving Sepsis Campaign

On the contrary, the early multimodal vasopressor therapy may overexpose the patient to AVP (or other vasopressors), and this may be possibly harmful [5] and also cost-ineffective. The main challenge remains to readily identify patient profiles during the early phase of resuscitation. Some authors have proposed various biomarkers linked to vasopressor response and outcomes in septic shock [1]. From a pragmatic perspective, we suggest considering the kinetics of NE dose increment. Basically, two dose requirement profiles can be observed in patients at the bedside (Fig. 1). A “refractory” profile, which corresponds to the need of an exponential increase in NE doses, and a “controlled” profile with progressive increase in NE dose up to a plateau do not reach toxic levels of NE.

As depicted in Fig. 1, both profiles will similarly overshoot the threshold of 0.5 µg/kg/min of NE dose and should trigger the infusion of AVP on top of NE in a stepwise approach. In the refractory profile, the earlier the AVP initiation, the greater chance of avoiding skyrocketing doses of NE and exposing the patient to harmful NE doses. In the “controlled” profile, adding AVP at the NE threshold of 0.5 µg/kg/min may not be necessary.

To date, clinical trials aiming at identifying which patients could benefit the most of early association of NE + AVP are lacking and are urgently needed. We believe that catecholamine dose requirements should be part of a tailored approach.

## Data Availability

The dataset supporting the conclusions of this article is included within the article.
